# Diversity of clonal complex 22 methicillin-resistant *Staphylococcus aureus* isolates in Kuwait hospitals

**DOI:** 10.3389/fmicb.2022.970924

**Published:** 2022-08-04

**Authors:** Samar S. Boswihi, Tina Verghese, Edet E. Udo

**Affiliations:** Department of Microbiology, Faculty of Medicine, Kuwait University, Kuwait City, Kuwait

**Keywords:** CC22-MRSA, genotypes, antibiotic resistance, *spa* typing, DNA microarray

## Abstract

CC22-MRSA is a major MRSA lineage that is widely reported globally. To characterize CC22-MRSA for trends in antibiotic resistance and emergence of variants, a total of 636 CC22 isolates identified by DNA microarray in 2016 (*n* = 195), 2017 (*n* = 227) and 2018 (*n* = 214) were investigated further using staphylococcal protein A (*spa*) typing and multilocus sequence typing. The isolates belonged to 109 *spa* types dominated by t223 (*n* = 160), t032 (*n* = 60), t852 (*n* = 59), t005 (*n* = 56) and t309 (*n* = 30) and 10 sequence types (STs) dominated by ST22 (85.5%). Genotypes CC22-MRSA-IV [tst1^+^]; CC22-MRSA-IV UK-EMRSA-15/Barnim EMRSA variants, CC22-MRSA-IV [PVL^+^], CC22-MRSA-IV [tst1^+^/PVL^+^] and CC22-MRSA-IV + V constituted >50% of the isolates. An increase from 2016 to 2018 were shown in isolates belonging to *spa* types t223 (43 to 62), t032 (18 to 27) and t309 (10 to 15) and genotypes CC22-MRSA-IV [tst1^+^] (89 to 102), CC22-MRSA-IV + V (12 to 30) and CC22-MRSA-IV [tst1^+^/PVL^+^] (12 to 22). Ninety-nine CC22-MRSA isolates were multi-resistant to three or more antibiotic classes with 76.7% of them belonging to CC22-MRSA-IV [PVL^+^] and CC22-MRSA-IV [tst1^+^/PVL^+^]. The study revealed an ongoing domination of the CC22-MRSA-[tst1^+^] genotype and the emergence of new clones bearing SCC*mec* IV + V and multiply resistant variants.

## Introduction

Methicillin-resistant *Staphylococcus aureus* (MRSA) is a major cause of healthcare-acquired as well as community-acquired infections in many parts of the world (Monecke et al., 2011; Tong et al., 2015; Lee et al., 2018). The ability of MRSA strains to cause infections is associated with the possession of a wide array of virulence factors that allow them to adhere to and colonize the cell surfaces, avoid the immune system, and have toxic effect on the host. These virulence determinants include extracellular and cell-associated factors, such as biofilm, capsule, coagulase, clumping factor, lipase, protein A and multiple toxins including staphylococcal enterotoxins (SEs) and toxic shock syndrome toxin 1 (TSST-1) (Tong et al., 2015; Lee et al., 2018; Gholami et al., 2019; Shahmoradi et al., 2019). Some strains of *S. aureus* also secrete leukocidins such as Panton-valentine leucocidin, and arginine catabolic mobile element (ACME), which destroy and inhibit the production of leukocytes therefore helping the bacterium to avoid the host’s immune response (Tong et al., 2015).

The application of molecular typing methods to investigate the clonal distribution of MRSA isolates have shown that MRSA isolates are diverse and belong to several clonal complexes (CCs) (Lee et al., 2018). However, most MRSA infections are still caused by epidemic MRSA (EMRSA) clones belonging to a limited number of clonal complexes (CC) that include CC5, CC8 (including ST239), CC22, CC30 and CC45 that have emerged in different countries due to the acquisition of different SCC*mec* elements by successful methicillin susceptible *S. aureus* clones (Enright et al., 2000; Ghebremedhin et al., 2007; Monecke et al., 2011; Lee et al., 2018).

Methicillin-resistant *S. aureus* (MRSA) clonal complex 22 (CC22-MRSA), also known as UK EMRSA-15, is an epidemic MRSA clone that was discovered in England in the early 1990s (Kerr et al., 1990; Richardson and Reith, 1993; O’Neil et al., 2001). Since the initial report in England, CC22-MRSA has spread to become the leading cause of bloodstream infections in the UK’s healthcare systems (Johnson et al., 2001; Boakes et al., 2011) and in other European countries including Ireland, Germany, Denmark, Belgium, Spain, Portugal, Malta, Sweden (Witte et al., 2001; Ghebremedhin et al., 2007; Monecke et al., 2011, 2017; Holtfreter et al., 2016), India (Manoharan et al., 2012; Shambat et al., 2012; Dhawan et al., 2015), Turkey (Oksuz et al., 2013), Singapore (Hsu et al., 2007), Australia (Pearman et al., 2001), and New Zealand (Monecke et al., 2011). It has also been reported in the Middle East including Palestine (Biber et al., 2012; Al Laham et al., 2015), Jordan (Aqel et al., 2015), Lebanon (Tokajian et al., 2010; Harastani et al., 2014), Kuwait (Udo et al., 2006, 2016), Saudi Arabia (Senok et al., 2016), Qatar (El-Mahdy et al., 2014) and United Arab Emirates (Boswihi et al., 2018; Senok et al., 2020a).

Recent studies have shown that CC22-MRSA lineage consists of diverse genotypes that differ in the carriage of antibiotic resistance determinants, SCC*mec* elements, and toxins including Panton–Valentine leukocidin (PVL) and toxic shock syndrome toxin-1 (TSST-1) (Monecke et al., 2011; Senok et al., 2016; Udo et al., 2016).

CC22-MRSA isolates were first detected in Kuwait in 2001 and consisted mostly of the UK EMRSA-15 variant (Udo et al., 2006). Since then, CC22-MRSA have been isolated consistently with novel variants reported among isolates obtained from patients in Kuwait hospitals (Senok et al., 2016; Udo et al., 2016). Molecular typing of CC22-MRSA obtained in 2010 using pulsed-field gel electrophoresis, *spa* typing, multilocus sequence typing and DNA microarray analysis identified three variants with the UK EMRSA-15/Middle Eastern variant [tst1^+^] as the dominant CC22-MRSA genotype with only three isolates belonging to the UK EMRSA-15 variant (Udo et al., 2016). The isolates obtained in 2010 belonged to a single sequence type (ST22) and 10 *spa* types with t223 (51.3%), t852 (13.5%), t032 (8.1%) and t790 (8.1%) as the common *spa* types (Udo et al., 2016).

The prevalence of CC22-MRSA has continued to increase in the Arabian Gulf countries in recent years. A recent study by Senok et al. (2016) reported six variants of CC22-MRSA in the region with the tst1^+^-CC22-MRSA-IV isolates belonging to t223 as the dominant variant and the UK EMRSA-15 isolates belonging to t032 detected sporadically.

As the number of CC22-MRSA isolates obtained from patients in Kuwait hospitals has continued to increase by year, this study was initiated to investigate the CC22-MRSA isolates obtained between 2016 and 2018 to ascertain trends in their genetic backgrounds, antibiotic resistance and virulence characteristics.

## Materials and methods

### Bacterial strain

The MRSA isolates used in this study were obtained as part of routine diagnostic microbiology investigations. The MRSA isolates were identified using traditional diagnostic bacteriological methods including Gram stain, growth on Mannitol Salt Agar, positive DNAse and tube coagulase tests. The isolation and identification of the isolates were performed in the diagnostic microbiology laboratories where initial antibiotic susceptibility testing was also performed with VITEK (bioMérieux, Marcy l’Etoile, France). Pure cultures of isolates on blood agar plates were submitted to the Gram-Positive Bacteria Research Laboratory, located in the department of Microbiology, Faculty of Medicine, Kuwait University, where the isolates were retested for purity and preserved in 40% glycerol (v/v in brain heart infusion broth) at −80°C for further analysis. Each isolate was from a single patient. The isolates were cultured from different clinical samples including nasal swabs (*n* = 234; 36.8%), sputum (*n* = 21; 3.3%), tracheal aspirates (*n* = 25; 4%), throat swabs (*n* = 17; 2.7%), pus (*n* = 53; 8.3%), wound (*n* = 46; 7.2%) and skin (*n* = 51; 8%). The remaining 116 isolates were collected from groin (*n* = 35; 5.5%), blood (*n* = 17; 2.6%), urine (*n* = 15; 2.3%), eye swabs (*n* = 12; 1.8%), ear swabs (*n* = 13; 2.0%), axilla (*n* = 10; 1.5%), high vaginal swab (*n* = 8; 1.2%) and fluid (*n* = 6; 0.9%). The clinical sources of 73 isolates were not provided. The isolates were recovered by two subcultures on brain heart infusion agar and incubate at 35 C before analysis.

### Antibiotic susceptibility testing

Antibiotic susceptibility testing was performed by the disk diffusion method (Clinical and Laboratory Standard Institute (CLSI), 2015) against benzyl penicillin (10 U), cefoxitin (30 μg), kanamycin (30 μg), mupirocin (200 and 5 μg), gentamicin (10 μg), erythromycin (15 μg), clindamycin (2 μg), chloramphenicol (30 μg), tetracycline (10 μg), trimethoprim (2.5 μg), fusidic acid (10 μg), rifampicin (5 μg). Minimum inhibitory concentration for cefoxitin, vancomycin, teicoplanin and linezolid were determined with Etest strips (bioMérieux, Marcy l’Étoile, France) according to the manufacturer’s instructions. *S. aureus* strains ATCC 25923 and ATCC 29213 were used as quality control strains for the disk diffusion and MIC determination, respectively. The Dtest was used to test for inducible resistance to clindamycin.

### Molecular typing methods

#### *Spa* typing

DNA isolation and purification were performed as described previously by Boswihi et al. (2020a). *Spa* typing was performed using protocol and primers published previously (Harmsen et al., 2003). The PCR protocol consisted of an initial denaturation at 94°C for 4 min, followed by 25 cycles of denaturation at 94°C for 1 min, annealing at 56°C for 1 min, and extension for 3 min at 72°C, and a final cycle with a single extension for 5 min at 72°C. Five μl of the PCR product was analyzed by 1.5% agarose gel electrophoresis to confirm amplification. The amplified PCR product was purified using Micro Elute Cycle-Pure Spin kit (Omega Bio-Tek, Inc., United States) and the purified DNA was then used for sequencing PCR. The sequencing PCR product was then purified using DyeEx 2.0 Spin Kit (Qiagen, United States). The Purified DNA was sequenced in an automated 3130 × 1 genetic analyzer (Applied Biosystem, USA). The sequenced *spa* gene was analyzed using the Ridom Staph Type software available at http://www.ridom.de/staphtype.

### SCC*mec* subtyping

The SCC*mec* types of the isolates were extracted from results of DNA Microarray analysis. The subtyping of SCC*mec* IV (IVa, IVb, IVc, IVg, IVh) was performed as described previously by Zhang et al. (2005) and Milheiriço et al. (2007). The PCR products were separated by agarose gel electrophoresis using 2% (w/v) agarose in Tris-EDTA buffer.

### Multilocus sequence typing

MLST was performed on representative isolates selected on the basis of *spa* types. Primers and PCR protocols was performed as described by Enright et al. (2000). Sequence types (STs) were obtained by determining the allele number for the seven housekeeping genes as described by Jolley et al. (2018).

### DNA microarray

DNA microarray analysis was performed using the Identibac *S. aureus* genotyping kit 2.0 and the ArrayMate reader (Alere Technology, Jena, Germany) as described previously by Monecke et al. (2008). The DNA microarray analysis was used for the simultaneous detection of SCC*mec* types, antibiotic resistance genotypes and virulence related genes, including PVL, genes encoding species markers, and to allocate clonal complex (CC). *S. aureus* genotyping array is presented in an ArrayStrip format which contains 336 probes printed onto an array located in the bottom of the ArrayStrip. MRSA isolates were grown on blood agar plates at 35°C overnight. DNA extraction of the overnight culture was performed as described by the manufacturer using Identibac *S. aureus* genotyping kit 2.0 (Alere, GmbH, Germany). Linear amplification of the purified DNA was performed in a total of 10 μl of the reaction volume containing 4.9 μl of B1 (Labeling Buffer/Master Mix), 0.1 μl of B2 (Labeling Enzyme), and 5 μl of the purified DNA. The PCR protocol consisted of an initial denaturation for 5 min at 96°C, followed by 50 cycles of denaturation for 60 s at 96°C, annealing for 20 s at 50°C, and extension for 40 s at 72°C. Hybridization and washing of the labeled arrays were performed as previously described (Monecke et al., 2008). The array was scanned using the ArrayMate reader (CLONDIAG, Alere, Germany) and the image of the arrays was recorded and analyzed using IconoClust software plug-in (CLONDIAG). The result was interpreted as negative, positive, or ambiguous by the software.

## Results

DNA microarray analysis performed on 5,223 MRSA isolates cultured from different clinical samples between 2016 and 2018 revealed that 636 (12.1%) of the MRSA isolates obtained in 2016 (*n* = 195), 2017 (*n* = 227) and 2018 (*n* = 214) belonged to clonal complex 22 (CC22-MRSA). The CC22-MRSA isolates were investigated further for their resistance to antibacterial agents, genotypes and virulence determinants.

The CC22-MRSA isolates were susceptible to vancomycin (MIC ≤2 μg/ml), teicoplanin (MIC ≤2 μg/ml), linezolid (MIC ≤4 μg/ml) and rifampicin but were resistant to trimethoprim (*n* = 447; 70.3%), ciprofloxacin (*n* = 273; 42.9%), kanamycin (*n* = 211; 33.2%), gentamicin (*n* = 208; 32.7%), erythromycin (*n* = 194; 30.5%), fusidic acid (*n* = 50; 7.9%), tetracycline (*n* = 34; 5.3%) and high-level resistance to mupirocin (*n* = 34; 5.3%). Inducible clindamycin resistance was detected in 111 isolates (17.4%), while 80 (12.6%) isolates expressed constitutive resistance.

### SCC*mec* typing and subtyping

The SCC*mec* types were obtained from the DNA microarray results. Three SCC*mec* types were associated with the CC22-MRSA isolates. These were SCC*mec* type IV (*n* = 559), SCC*mec* types V (*n* = 5) and VI (*n* = 4) with SCC*mec* type IV clearly the most common SCC*mec* type. Sixty-one (9.6%) of isolates carried both SCC*mec* elements IV and V (SCC*mec* IV + V).

The subtyping of isolates carrying SCC*mec* IV was performed by multiplex PCR. The results showed subtype IVa, detected in 286 (45%) isolates, was the most common subtype followed by subtype IVh that was detected in 55 (8.6%) isolates. No SCC*mec* IV subtypes were identified for 217 isolates.

### *Spa* typing

One hundred and nine different *spa* types were identified among the CC22-MRSA isolates. Most of the isolates belonged to *spa* types t223 (*n* = 160), t032 (*n* = 60), t852 (*n* = 59), t005 (*n* = 56) and t309 (*n* = 30) which constituted 56.4% of the CC22-MRSA isolates. The remaining 113 *spa* types were detected in less than 10 isolates each. *Spa* types detected in fewer than 10 isolates were considered sporadically detected. The complete list of the *spa* types is presented in Supplementary Table S1.

**Figure 1:**
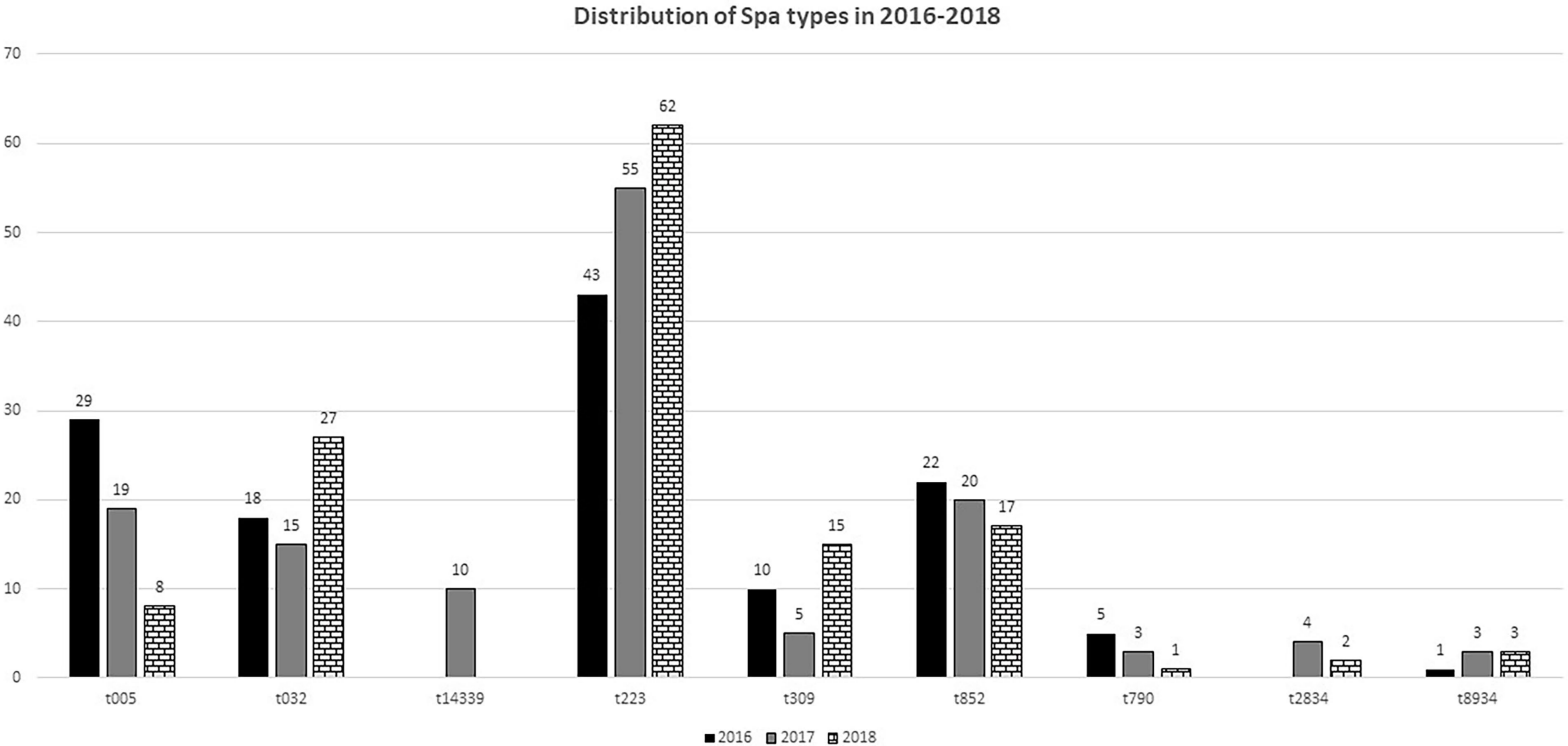
Distribution of the common *spa* types in CC22-MRSA isolates in 2016–2018.

The distribution of some of the common *spa* types varied over the 3 years. The results presented in Figure 1, shows that isolates of *spa* type t223, increased from 43 isolates in 2016 to 62 in 2018. The number of t032 and t309 isolates decreased in 2017 but increased in 2018 (Figure 1). On the other hand, the number of t852 isolates decreased from 22 in 2016 to 17 in 2018. Similarly, the number of t005 isolates decreased from 29 in 2016 to eight in 2018. Some sporadic *spa* types were isolated only in certain years. For example, t14339 isolates were only detected in 2017 but not detected in 2018 (Figure 1).

The results showed that SCC*mec* IVa, the dominant subtype, was widely distributed among the different *spa* types. It was present in 110 of 160 t223 isolates and in 21 of 30 t309 isolates. SCC*mec* IVh subtype was detected in most of t032 (24/60) isolates.

### Distribution of MLST sequence types

Ninety-seven CC22-MRSA isolates representing different *spa* types were selected for MLST to determine their sequence types. The 97 isolates belonged to 10 sequence types (STs) with the majority belonging to ST22 (*n* = 83). The remaining nine STs were ST1037 (*n* = 2), ST1082 (*n* = 2), ST2286 (*n* = 2), ST2371 (*n* = 2), ST244 (*n* = 1), ST737 (*n* = 1), ST2124 (*n* = 1), and ST4671 (*n* = 1). A new sequence type, ST5868, was identified in two isolates.

### Prevalence and molecular characteristics of CC22-MRSA isolates in 2016–2018

DNA microarray analysis classified the CC22-MRSA isolates into 13 different genotypes as summarized in Table 1. Most of the isolates (*n* = 305; 48%) were CC22-MRSA-IV [tst1^+^]. This was followed by 69 isolates consisting of CC22-MRSA-IV [fnbB^+^] (*n* = 35), CC22-MRSA-IV [fnbB-sec/l^+^] (*n* = 22) and CC22-MRSA-IV [fnbB-sec/l−] (*n* = 12) classified as the UK-EMRSA-15/Barnim EMRSA variants. The remaining 262 isolates belonged to CC22-MRSA-IV [PVL^+^] (*n* = 136), CC22-MRSA-IV + V (*n* = 61), CC22-MRSA-IV [tst1^+^/PVL^+^] (*n* = 47), CC22-MRSA-IV + V [PVL^+^] (*n* = 7), CC22-MRSA-V [fusC] (*n* = 3), CC22-MRSA-[VI + fus] (*n* = 4 isolates), CC22-MRSA-V (*n* = 2), CC22-MRSA-[IV + fus + ccrAB4] (*n* = 1) and CC22-MRSA-IV [Q6GD50^+^], UK-EMRSA-15/Maltese variant (*n* = 1). The characteristics of the genotypes are presented below.

**Table 1:** Antibiotic resistance profile among CC22 isolates.

CC22 Genotypes	Multi-resistance	#	Non-multi-resistance	#
CC22-MRSA-IV [tst1^+^/PVL^+^] (*n* = 47)	*aacA-aphD*, *dfrS1*, *erm*(*C*)	26	*aacA-aphD*, *dfrS1*	14
	*erm*(*C*), *aacA-aphD*, *dfrS1*, *tet(K*)	1	*erm*(*C*), *dfrS1*	2
			*dfrS1*	2
			*erm*(*C*)	1
			*Susceptible to non-beta-lactams*	1
CC22-MRSA-IV [fnbB-,sec/l−], UK-EMRSA-15/Barnim EMRSA (*n* = 12)			*dfrS1*	6
			*erm*(*C*)	3
			*vga*(*A*)	1
			*erm*(*C*), *vga*(*A*)	1
			*Susceptible to non-beta-lactams*	1
CC22-MRSA-IV [fnbB-, sec/l^+^], UK-EMRSA-15/Barnim EMRSA (*n* = 22)			*erm*(*C*)	18
			*Susceptible to non-beta-lactams*	4
CC22-MRSA-IV [fnbB^+^], UK-EMRSA-15/Barnim EMRSA (*n* = 35)	*aacA-aphD*, *aadD*, *dfrS1*, *erm*(*C*)	2	*aacA-aphD*, *aadD*, *dfrS1*	10
	*aacA-aphD*, *dfrS1 erm*(*C*)	1	*aacA-aphD*, *aadD*, *erm*(*C*)	4
	*aacA-aphD*, *aadD*, *dfrS1 erm*(*C*), t*et*(*K*)	1	*aacA-aphD*, *aadD*	2
			*aacA-aphD*, *dfrS1*	1
			*aacA-aphD*	1
			*erm*(*C*)	3
			*Susceptible to non-beta-lactams*	10
CC22-MRSA-IV [PVL^+^] (*n* = 136)	*aacA-aphD*, *aadD*, *dfrS1*, *erm*(*C*)	34	*aacA-aphD*, *aadD*, *dfrS1*	49
	*aacA-aphD*, *dfrS1*, *erm*(*C*)	4	*aacA-aphD*, *aadD*, *erm*(*C*)	7
	*aacA-aphD*, *aadD*, *dfrS1*, *erm*(*C*), *tet*(*K*)	2	*aacA-aphD*, *aadD*	10
	*aacA-aphD*, *aadD*, *dfrS1*, *erm*(*C*), *fusB*	2	*aacA-aphD*, *dfrS1*	6
	*aacA-aphD*, *aadD*, *dfrS1*, *fusB*	1	*aadD*, *erm*(*C*)	5
	*aacA-aphD,aadD erm(C),cat*	1	*dfrS1, erm(C)*	3
	*aacA-aphD,aadD,dfrS1, erm(C),fusB,mupA, cat, qacA*	1	*aacA-aphD, erm(C)*	2
	*aacA-aphD, aadD,dfrS1, erm(C),vga(A)*	1	*aacA-aphD,vga(A)*	1
	*msr(A),mph(C),aacA-aphD,aadD,aphA3,sat,dfrS1*	1	*erm(C), dfrS1*	1
	*aacA-aphD,aadD,dfrS1,vga(A)*	1	*aadD, dfrS1*	1
	*aadD,dfrS1, erm(C)*	1	*aadD*	1
			*dfrS1*	1
**CC22 Genotypes**	**Multi-resistance**	#	**Non-multi-resistance**	#
CC22-MRSA-IV [tst1^+^], UK-EMRSA-15/Middle Eastern Variant (*n* = 305)	*dfrS1, aacA-aphD, erm(C)*	7	*dfrS1*	222
	*dfrS1, erm(C),te(tK)*	2	*dfrS1, tet(K)*	12
			*dfrS1, erm(C)*	9
			*dfrS1, aacA-aphD*	5
			*dfrS1,cat*	1
			*dfrS1, erm(C),cat*	1
			*dfrS1, erm(C),vga(A)*	1
			*dfrS1,vga(A)*	2
			*tet(K)*	2
			*erm(C)*	2
			*vga(A)*	1
			*Susceptible to non-beta-lactams*	38
CC22-MRSA-IV + V (*n* = 61)	*erm(C), mupA, sat*	1	*erm(C),mupA*	24
	*erm(C), dfrS1, tet(M), fexA*	1	*erm(C)*	12
			*mupA*	11
			*fusC*	5
			*dfrS1*	1
			*Susceptible to non-beta-lactams*	6
CC22-MRSA-IV + V [PVL^+^] (*n* = 7)	*erm(C), aacA-aphD, dfrS1, mupA tet*(*K*)	1	*aacA-aphD, aadD,fusC*	1
	*erm(C), aacA-aphD, dfrS1, tet*(*K*)	1	*fusC*	1
	*erm(C),aacA-aphD, dfrS1, vga(A)*	1	*Susceptible to non-beta-lactams*	1
	*aacA-aphD, aadD,dfrS1, vga(A)*	1		
CC22-MRSA-[VI + fus] (*n* = 4)	*vga(A), dfrS1, fusC*	2	*fusC*	1
			*dfrS1*	1
CC22-MRSA-V (n = 2)			*tet*(*K*)	2
CC22-MRSA-V [fusC^+^] (*n* = 3)			*fusC*	3
CC22-MRSA-IV [Q6GD50^+^], UK-EMRSA-15/Maltese Variant (*n* = 1)			*fusC*	1
CC22-MRSA-[IV + fus + ccrAB4] (*n* = 1)	*erm(C), aacA-aphD, dfrS1*	1		

aacA-aphD, aminoglycoside adenyl−/phoshotransferase; aadD, aminoglycoside adenyl transferase; aphA3, aminoglycoside phosphotransferase; cat, chloramphenicol acetyl transferase; fexA, chloramphenicol/florfenicol exporter; dfrS1, dihydrofolate reductase mediating trimethoprim resistance; erm(C), rRNA methyltransferase (C); msr(A), macrolide efflux pump; mph(C), macrolide phosphotransferase; vga(A), ABC transporter conferring resistance to streptogramin A and related compounds; fusC, fusidic acid resistance gene (Q6GD50); fusB, fusidic acid resistance gene (=far1); mupA, isoleucyl-tRNA synthethase associated with mupirocin resistance; tet (*K*), tetracycline efflux protein; tet(M), ribosomal protection protein associated with tetracycline resistance; sat, streptothricin acetyltransferase; and qacA, multidrug efflux protein A.

### CC22-MRSA-IV [tst1^+^], UK-EMRSA-15/Middle Eastern variant

**Figure 2:**
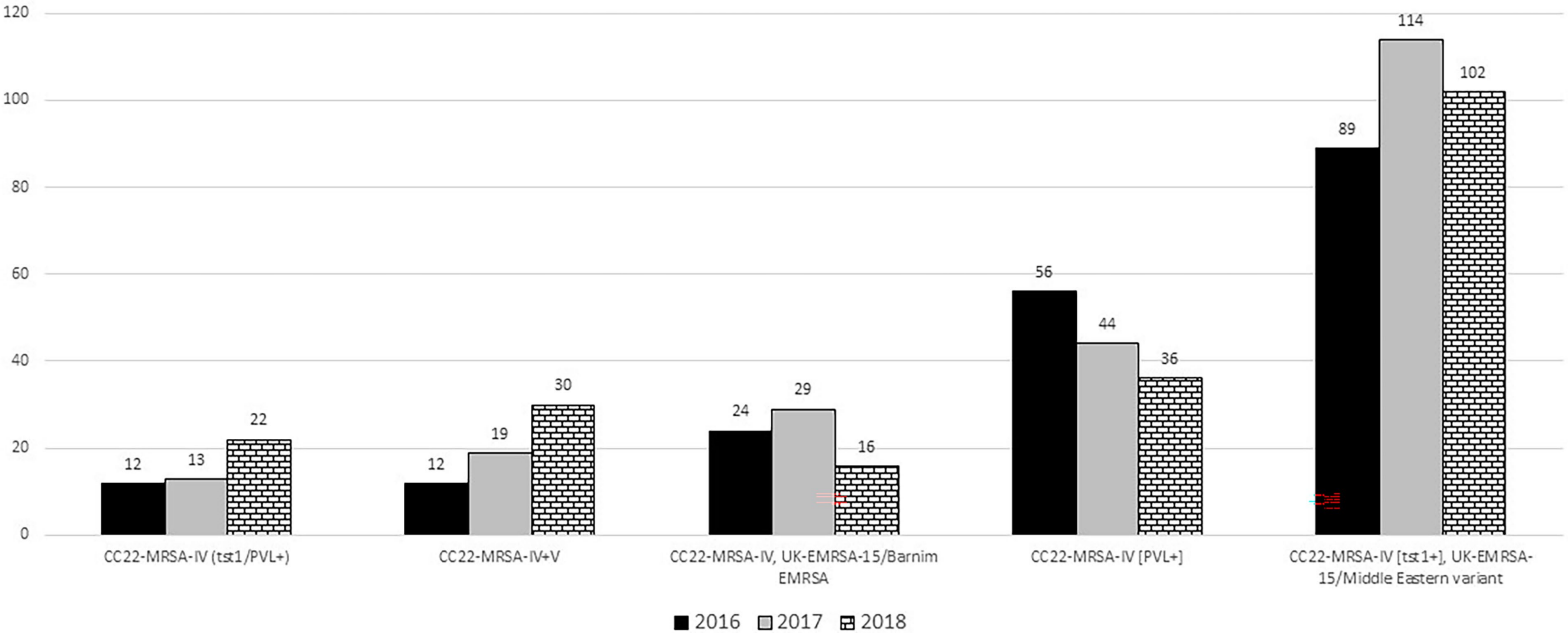
Distribution of the common CC22-MRSA genotypes in 2016–2018.

The number of CC22-MRSA-IV [tst1^+^] isolates increased from 89 isolates in 2016 to 114 in 2017 and declined slightly to 102 isolates in 2018 (Figure 2). *Spa* typing showed that the isolates belonged to 62 *spa* types with t223 as the most common detected in 134 (44.0%) of the 305 isolates. Most of the 38 isolates selected for MLST belonged to ST22 (*n* = 34), while the remaining isolates belonged to ST1082 (*n* = 2), ST1037 (*n* = 1) and ST244 (*n* = 1). Two hundred and nineteen of the isolates carried SCC*mec* IVa, and one isolate carried SCC*mec* IVh. The rest of the isolates carried SCC*mec* IV without subtypes.

All of the CC22-MRSA-IV [tst1^+^] isolates were positive for *tst1* and the enterotoxin gene cluster (egc; *seg*, *sel-i*, *sel-m*, *sel-n*, *sel-o*, *sel-u*). Trimethoprim resistance mediated by *dfrS1* was detected in 222 isolates. Ten isolates were multiply resistant to gentamicin, kanamycin, erythromycin, clindamycin, tetracycline and chloramphenicol encoded by *aacA-aphD*, *aadD*, *erm*(*C*), *tet*(*K*) and *cat*, respectively (Table 1).

### CC22-MRSA-IV, UK-EMRSA-15/Barnim EMRSA

The 69 UK-EMRSA-15/Barnim EMRSA isolates consisted of three variants: CC22-MRSA-IV [fnbB-sec/l−] (*n* = 12), CC22-MRSA-IV [fnbB-sec/l^+^] (*n* = 22) and CC22-MRSA-IV [fnbB^+^] (*n* = 35). The distribution of the UK-EMRSA-15/Barnim EMRSA variants from 2016 to 2018 is shown in Figure 2. Their numbers increased from 24 in 2016 to 29 in 2017 then declined to 16 in 2018. Twenty-five different *spa* types including t852 (*n* = 15), t032 (*n* = 13) and t790 (*n* = 9) were associated with the UK-EMRSA-15/Barnim EMRSA variant. The t032 isolates were distributed among CC22-MRSA-IV [fnbB-, sec/l−] (*n* = 5) and CC22-MRSA-IV [fnbB-, sec/l^+^] (*n* = 8). The 15 t852 and seven t790 isolates were distributed among CC22-MRSA-IV [fnbB^+^]. MLST revealed that the 16 representative isolates belonged to ST22 (*n* = 14), ST1037 (*n* = 1) and ST2371 (*n* = 1). Nineteen isolates carried SCC*mec* subtype IVa, whereas 13 isolates carried SCC*mec* subtype IVh. The distribution of the SCC*mec* subtypes among the three UK-EMRSA-15/Barnim EMRSA variants is shown in Supplementary Table S2.

The UK-EMRSA-15/Barnim EMRSA variant isolates were positive for egc encoding genes but varied in the carriage of *sea*, *seb*, *sec*, *sed* and *sel* (Supplementary Table S2). The isolates expressed varied resistance to erythromycin, trimethoprim, gentamicin, kanamycin and tetracycline mediated by *erm*(*C*), *dfrS1*, *aacA-aphD*, *aadD* and *tet*(*K*) respectively (Table 1). Four of the isolates expressed multiresistance to erythromycin, gentamicin, kanamycin, trimethoprim and tetracycline encoded by *erm*(*C*), *aacA-aphD*, *aadD*, *dfrS1*, *tet*(*K*) respectively (Table 1).

### CC22-MRSA-IV [PVL^+^]

The prevalence of CC22-MRSA-IV [PVL^+^] genotype decreased from 56 isolates in 2016 to 44 isolates in 2017 and 36 isolates in 2018 (Figure 2). Thirty *spa* types were identified among the 136 PVL-positive CC22-MRSA-IV isolates with t852 (*n* = 42), t005 (*n* = 31), t223 (*n* = 7), t2518 (*n* = 6) and t902 (*n* = 5) constituting 67.0% of the isolates (Supplementary Table S2). Thirteen of the 16 representative isolates selected for MLST belonged to ST22. The other sequence types, ST4671, ST2286 and ST2371, occurred in single isolates. Thirty isolates carried SCC*mec* subtypes IVa (*n* = 15) and IVh (*n* = 15). No SCC*mec* IV subtype were identified in 14 isolates.

The isolates varied in the carriage of genes for Staphylococcal enterotoxin genes and toxic shock syndrome toxin. In addition to the genes for PVL, six of the isolates were positive for *tst1*. Other isolates were also positive for *sec* (*n* = 5), *sel* (*n* = 5), *sek* (*n* = 1) and *seq* (*n* = 1). Eighty-seven (64%) of the PVL-positive isolates were resistant to one or two antibiotics while 49 (36%) isolates expressed multiple resistance to antibiotics (Table 1).

### CC22-MRSA-IV + V

The numbers of isolates belonging to CC22-MRSA-IV + V genotype increased from 12 in 2016 to 30 in 2018. Of the 61 isolates, 41 (67.2%) belonged to *spa* type t032, 12 isolates belonged to eight different *spa* types, and nine isolates could not be assigned to a *spa* type (Supplementary Table S2). All six representative isolates selected for MLST belonged to ST22. Seven isolates carried SCC*mec* subtype IVa and 24 isolates carried SCC*mec* subtype IVh.

All 61 isolates were positive for *egc*. The other toxin genes detected in these isolates were *sec* (*n* = 26), *sel* (*n* = 32), *sea* (*n* = 1), *seb* (*n* = 2) and *tst1* (*n* = 9). Thirty-eight of the CC22-MRSA-IV + V isolates were resistant to erythromycin and clindamycin mediated by *erm*(*C*), while 36 and five isolates were resistant to high-level mupirocin and fusidic acid mediated by *mupA* and *fusC, respectively.* One isolate was resistant to erythromycin, clindamycin, trimethoprim, tetracycline and chloramphenicol mediated by *erm*(*C*), *dfrS1*, *tet*(*M*) and *fexA*, respectively (Table 1).

### CC22-MRSA-IV [tst1^+^/PVL^+^]

Isolates identified as CC22-MRSA-IV [tst1^+^/PVL^+^] increased from 12 in 2016 to 22 in 2018. Ten *spa* types were identified among the isolates with t005 and t309 detected in 16 and 10 isolates, respectively. The other *spa* types occurred in single isolates. The 12 representative isolates selected for MLST belonged to ST22 (*n* = 11) and ST2286 (*n* = 1). Twenty-three isolates carried SCC*mec* IVa. The rest of the isolates carried SCC*mec* IV without subtypes.

The isolates were all positive for PVL, *tst1* and *egc* but varied in the carriage of *sec* and *sel.* Twenty isolates were resistant to one or two antibiotic classes whereas 27 isolates were multi-resistant to erythromycin and clindamycin, gentamicin, trimethoprim, and tetracycline mediated by *erm*(*C*), *aacA-aphD*, *dfrS1*, and *tet*(*K*) respectively (Table 1).

### Sporadic CC22-MRSA genotypes

The Six CC22-MRSA genotypes described as sporadic were detected in less than 10 isolates during the study period. The sporadic genotypes were CC22-MRSA-[VI + fus] (*n* = 4), CC22-MRSA-IV + V [PVL^+^] (*n* = 7), CC22-MRSA-V [fusC] (*n* = 3), CC22-MRSA-V (*n* = 2), CC22-MRSA-[IV + fus + ccrAB4] (*n* = 1) and CC22-MRSA-IV [Q6GD50], UK-EMRSA-15/Maltese variant (*n* = 1).

The CC22-MRSA-[VI + fus] (*n* = 4) genotype was detected once in 2016 and 2018, and twice in 2017. All four isolates were positive for *tst1* and *egc* and were resistant to fusidic acid mediated by *fusC*. The isolates obtained in 2017 and 2018 were resistant to trimethoprim mediated by *dfrS1*. The four isolates belonged to two *spa* types and two sequence types, t16578/ST2124 (*n* = 1) and t8934/ST22 (*n* = 3).

The CC22-MRSA-IV + V [PVL^+^] (*n* = 7) genotype was detected in one, four and two isolates in 2016, 2017 and 2018, respectively. One isolate carried SCC*mec* subtype IVa while the remaining six isolates carried SCC*mec* IV with no subtypes. All seven isolates were positive for gene for PVL and *egc*. In addition, four isolates were positive for *tst1* and two isolates were positive for *sec* and *sel*. Four of the seven isolates were multiply resistant to gentamycin, kanamycin, trimethoprim, erythromycin and clindamycin, tetracycline, high-level mupirocin and virginamycin mediated by *aacA-aphD*, *aadD*, *dfrS1*, *erm*(*C*), *tet*(*K*), *mupA* and *vga*(*A*) respectively (Table 1).

CC22-MRSA-V [fusC^+^] (*n* = 3) genotype was detected once in 2017, twice in 2018 and not in 2016. All the three isolates belonged to *spa* type t223 and ST22. They were positive for *tst1*, egc and *fusC*.

The two CC22-MRSA-V isolates were detected in 2018 and belonged to *spa* type t2860, and sequence type ST737. Both were positive for *sea, egc* and *tet*(*K*).

The single Maltese ST22-MRSA-IV-[Q6GD50^+^]-t541 was detected in 2018. It was positive for *egc* and *fusC* and carried SCC*mec* subtype IVa. The single CC22-MRSA-[IV + fus + ccrAB4] isolate, detected in 2017, was positive for PVL, *tst1*, *egc*, *sec* and the antibiotic resistance genes, *erm*(*C*), *aacA-aphD* and *dfrS1* (Table 1). SCC*mec* IV subtype was not detected in this isolate.

## Discussion

This study has revealed changes in the clonal composition of CC22-MRSA isolates obtained in Kuwait hospitals in 2016, 2017 and 2018. CC22-MRSA constituted 12.1% of MRSA obtained during the study period, and belonged to 109 *spa* types, four SCC*mec* types (SCC*mec* types IV, V, VI and IV + V), 10 sequence types and 13 genotypes, which represents a substantial increase in the numbers and genetic diversity of the CC22-MRSA lineage in Kuwait hospitals. In comparison, our previous study on the composition of CC22-MRSA in 2010 revealed a population of isolates that belonged to a single SCC*mec* type (SCCmec IV), 10 *spa* types, a single sequence type (ST22) and three genotypes (Udo et al., 2016).

The population of CC22-MRSA isolates increased from 195 isolates in 2016 to 214 in 2018 compared with the 37 isolates that was collected in 2010 (Udo et al., 2016). The observed expansion in the proportion of CC22-MRSA isolates in Kuwait hospitals mimics reports from some Asian and European countries where the numbers of CC22-MRSA isolates are also high (Marchese et al., 2009; Conceição et al., 2013; Sunagar et al., 2016; Niek et al., 2019). Similarly, recent studies in China reported increase in the population of ST22-MRSA isolates among patients suffering from skin and soft tissue infections (Zhao et al., 2012; Xiao et al., 2019).

Besides the overall increase in the population of CC22-MRSA during the study period, there were changes in the numbers and types of SCC*mec* genetic elements. The detection of SCC*mec* types IV, V, VI, and IV + V in this study is in sharp contrast to the single SCC*mec* type (SCC*mec* IV) detected in our previous study in Kuwait (Udo et al., 2016) and in other Gulf Cooperation Council countries (Senok et al., 2016). CC22-MRSA isolates carrying SCC*mec* type V are rare and were only reported in a single isolate in the United Kingdom (Boakes et al., 2011) and in two isolates obtained in the Gaza strip (Al Laham et al., 2015) prior to this study. Therefore, the high number of SCC*mec* V-CC22-MRSA isolates in this study and in our previous report (Boswihi et al., 2018, 2020a) shows that it is expanding in Kuwait. The presence of different SCC*mec* types in the CC22-MRSA lineage suggests recent independent acquisition of the SCC*mec* elements.

*Spa* typing identified 109 *spa* types with *spa* types, t223 (*n* = 160; 25.1%), t032 (*n* = 60; 9.4%), t852 (*n* = 59; 9.2%), t005 (*n* = 56; 8.8%) and t309 (*n* = 30; 4.7%) as the dominant *spa* types. In contrast, only 10 *spa* types were associated with CC22-MRSA isolates in Kuwait in 2010 (Udo et al., 2016). However, although the numbers and types of *spa* types have increased since 2010 (Udo et al., 2016) t223 has remained the dominant *spa* type, followed by t852 and t032 among the CC22-MRSA isolates as was the case in 2010 (Udo et al., 2016). The t223 isolates are widely reported in many countries with a global frequency of 0.61% (http://spa.ridom.de/frequencies.shtml). The proportion of t309 isolates increased from one in 2010 to 15 in 2018, while t005 was not identified in CC22-MRSA isolates prior to this study in Kuwait hospitals. Therefore, t005 is an emerging *spa* type among CC22-MRSA isolates in Kuwait.

Although 10 sequence types were identified among the CC22-MRSA isolates in this study, most (85.5%) of the isolates belonged to ST22 with the rest of the isolates distributed among ST1037, ST1082, ST2286, ST2371, ST244, ST737, ST2124, ST4671 and the novel sequence type, ST5868. The dominance of ST22 is consistent with the sequence type associated with most CC22-MRSA reported in studies from different countries (Shore et al., 2010, 2012; Couto et al., 2015; Dhawan et al., 2015; Goudarzi et al., 2016; Sit et al., 2017; Udo and Al-Sweih, 2017; Firoozeh et al., 2020; Pomorska et al., 2021) indicating that CC22-MRSA has remained homogenous until now when characterized using MLST. However, the detection of nine other sequence types together with variations in *spa* and SCC*mec* types in this study signals an emerging genomic diversification in the CC22-MRSA lineage.

The *tst1*-positive CC22-IV-MRSA, also known as the Middle Eastern variant of UK-EMRSA-15 variant, was the leading genotype in this study. It was also the predominant genotype among CC22-MRSA isolates in Kuwait in 2010 (Udo et al., 2016), and in Egypt (Khairalla et al., 2017), Palestine (Al Laham et al., 2015), Jordan (Aqel et al., 2015) and UAE (Senok et al., 2020a). Some isolates belonging to *spa* type t032 and t852 that are not members of the Middle Eastern variant were found to also harbor *tst1* in this study. Furthermore, three new sequence types, ST244, ST1037, and ST1082 were associated with the *tst1*-positive CC22-IV-MRSA, UK EMRSA-15/Middle Eastern variant demonstrating the diversification of the CC22-MRSA lineage. Most of the *tst1*-positive CC22-IV-MRSA isolates in this study belonged to *spa* type t223 and were resistant to trimethoprim encoded by *dfrS1* similar to the isolates reported previously in Kuwait (Udo et al., 2016). Most (70%) of the t233 isolates in this study belonged to SCC*mec* subtype IVa, one isolate carried SCC*mec* IVh and the rest carried SCC*mec* IV without subtypes. However, the presence of *tst1* in an isolate with t032 carrying SCC*mec* IVh may suggest the acquisition of *tst1* by a UK ENRSA-15/Barnim MRSA clone rather than a UK EMRSA-15/Middle Eastern variant acquiring SCC*mec* IVh.

The CC22-MRSA-IV [PVL^+^] detected in 64.7% of the isolates was the second common genotype in this study. Although most of the isolates were non-multiresistant, 35.0% of the PVL^+^ isolates were multiresistant to erythromycin, clindamycin, gentamicin, kanamycin, trimethoprim, tetracycline, chloramphenicol, fusidic acid encoded by *erm*(*C*), *msr*(*A*), *mph*(*C*), *aacA-aphD*, *aadD*, *aphA3*, *dfrS1*, *tet*(*K*), *cat* and *fusB*, respectively (Table 1). Previously, most of the CC22-MRSA-IV [PVL^+^] isolates were associated with t852 (Udo et al., 2016), which is consistent with the observation in this study. In addition, isolates belonging to t005, t223, t309, t2518, t902 and other sporadic *spa* types have been identified with CC22-MRSA-IV [PVL^+^] in this study adding to the growing list of new CC22-MRSA variants in Kuwait. Although reported for the first time in Kuwait, CC22-MRSA-IV [PVL^+^]-t005 isolates have been seen in isolates obtained in England (Boakes et al., 2011), Ireland (Shore et al., 2014), Germany (Busche et al., 2018), Palestine (Al Laham et al., 2015) and Iran (Goudarzi et al., 2020) suggesting a recent importation into Kuwait.

The CC22-MRSA-IV, UK EMRSA-15/Barnim EMRSA variant was the third common CC22-MRSA variant in this study. It represents the early or classical UK-EMRSA-15 isolates reported by Richardson and Reith (1993) which are characterized by the carriage of enterotoxins, *sec*, *sel* and *egc* and the clindamycin and erythromycin resistance gene, *erm*(*C*) (Monecke et al., 2011). The numbers of UK EMRSA-15/Barnim EMRSA variant reported in this study shows a marked increase from the three isolates reported in 2010 (Udo et al., 2016). The CC22-MRSA-IV, UK EMRSA-15/Barnim EMRSA variants usually harbor SCC*mec* subtype IVh and belong to *spa* type t032 (Ghebremedhin et al., 2007; Boakes et al., 2011; Monecke et al., 2011; Couto et al., 2015; Holtfreter et al., 2016). However, in this study, only a proportion of the isolates belonged to t032 (*N* = 13) and harbored SCC*mec* IVh. The other CC22-MRSA-IV, UK EMRSA-15/Barnim EMRSA variants isolates belonged to other *spa* types including t852, t790, t223, harbored SCC*mec* IVa and others presented in Supplementary Table S2 did not have SCC*mec* IV subtypes. Similar to the results of this study, CC22-MRSA-IV/Barnim strains isolated in Germany also carried SCC*mec* IVa and t032 (Ghebremedhin et al., 2007). In contrast, most of the t032 CC22-MRSA reported by Boakes et al. (2011) from the UK carried SCC*mec* IVc whereas the t032 CC22-MRSA studied in Ireland by Shore et al. (2012) were dominated by SCC*mec* IVh suggesting that CC22-MRSA isolates acquired the SCC*mec* variants independently (Boakes et al., 2011). The results of this study shows that some of the isolates were related to the classical UK-EMRSA-15 variant and the Irish isolates carrying t032 and SCC*mec* IVh (Shore et al., 2012) while others were related to the German variant carrying SCC*mec* IVa, suggesting that CC22-MRSA-IV, UK EMRSA-15/Barnim EMRSA isolates in Kuwait were acquired from different routes. The CC22-MRSA-IV, UK EMRSA-15/Barnim EMRSA variants in this study also varied in the carriage of genes for fibronectin binding protein B (*fnbB*) and staphylococcal enterotoxin C (*sec*). The co-existence of these variants within the CC22-MRSA-IV, UK EMRSA-15/Barnim EMRSA variants provides additional evidence for the ongoing diversification of CC22-MRSA lineage in Kuwait hospitals.

The CC22-MRSA-IV + V is one of the novel variants that was not detected in Kuwait prior to 2016 but has increased from 2016 to 2018 (Figure 2). The isolates belonged to 10 *spa* types including t032 that is normally associated with the UK-EMRSA-15/Barnim variant. A few of the isolates (7/67) carried an additional gene for PVL resulting in the CC22-MRSA-IV + V [PVL^+^] variant. The presence of SCC*mec* IV + V elements and other combinations of SCC*mec* elements in the same isolate have previously been reported in MRSA isolates belonging to other sequence types obtained in India (Bhutia et al., 2015; Nagasundaram and Sistla, 2019). Although the report of this phenomenon is rare among MRSA isolates, it is more common in methicillin-resistant coagulase negative staphylococci (Mombach et al., 2007; Ternes et al., 2013; Al-Haqan et al., 2020). The presence of multiple SCC*mec* elements in a single strain has been explained by the acquisition of the SCC*mec* genetic elements on multiple occasions which increases the possibility of an integration of a new SCC*mec* element into an already existing one, giving rise to composite or mosaic SCC*mec* elements (Nagasundaram and Sistla, 2019). The presence of the SCC*mec* IV + V elements with diverse *spa* types support the multiple acquisition of genetic elements into a strain with an already existing genetic element.

Another interesting finding of this study is the increase in the number of CC22-MRSA isolates carrying the genes for PVL and *tst1* in the same strain. Although reported rarely in the literature, CC22-MRSA-IV [tst1^+^/PVL^+^] constituted 7.4% of the CC22-MRSA in this study. The number of CC22-MRSA-IV [tst1^+^/PVL^+^] isolates increased from 12 in 2016 to 22 in 2018 indicating its gradual expansion in Kuwait. The majority (57.7%; 27 isolates) of these isolates in this study were multi-resistant to erythromycin, clindamycin, gentamicin, trimethoprim, and tetracycline mediated by *erm*(*C*), *aacA-aphD*, *dfrS1*, *tet*(*K*) respectively (Table 1). Besides the report from Kuwait, five CC22-MRSA-IV [tst1^+^/PVL^+^] isolates were recovered from patients’ samples in Riyadh, Saudi Arabia in 2017 (Senok et al., 2019) and in the United Arab Emirates in 2018 (Senok et al., 2020b) confirming the recent introduction of the genotype into the Arabian Gulf countries.

The CC22-MRSA-IV [tst1^+^/PVL^+^] isolates have also been reported in swine and rhesus macaques’ monkeys in Nepal (Roberts et al., 2018, 2019, 2020). The Nepalese CC22-MRSA-IV [tst1^+^/PVL^+^] strains of animal origin were multiresistant with similar resistance profiles to our isolates (Roberts et al., 2018, 2019, 2020). It is interesting that the Rhesus monkey strains reported by Roberts et al. (2018) were also isolated between June 2015–June 2016, whereas the first isolates of CC22-MRSA-IV [tst1^+^/PVL^+^] in Kuwait were isolated between January and December 2016 (Boswihi et al., 2018, 2020b) and those from Riyadh, Saudi Arabia were isolated in 2017 confirming the recent evolution of this clone in both human and animal populations. Besides being detected in the CC22-MRSA genetic background, the tst1^+^/PVL^+^ combination was recently reported in the CC30-MRSA background (CC30-MRSA-VI + *fusC* [PVL^+^/tst1^+^]) (Boswihi et al., 2018) suggesting possible transmission by horizontal gene transfer.

The other genotypes identified in the current study were CC22-MRSA-[VI + fus], CC22-MRSA-V, CC22-MRSA-V [fusC], CC22-MRSA-IV[Q6GD50^+^] UK-EMRSA-15/Maltese variant and CC22-MRSA-[IV + fus + ccrAB4]. These were detected sporadically in small numbers and represent emerging variants of the CC22-MRSA lineage in Kuwait.

While most of the CC22-MRSA isolates in the current study were susceptible to most of the non-beta-lactam antibiotics like the classical epidemic clone UK-EMRSA-15/Barnim clone, a significant number of the current isolates were resistant to multiple antibiotics. The acquisition of multiple antibiotic resistance determinants and variants of the SCC*mec* elements may enhance their survival and spread.

The limitations of this study include the lack of information on the travel history of the patients that would explain the origin of some of the novel genotypes, and the absence of data on their prior antibiotic consumption which could explain the emergence of the multiple drug resistant genotypes.

In conclusion, the current study demonstrates an expansion of the CC22-MRSA isolates overtime, with the CC22-IV [tst1^+^] UK EMRSA-15/Middle Eastern variant remaining the most common genotype circulating in Kuwait hospitals. In addition, the study revealed the emergence of novel CC22-MRSA variants with double SCC*mec* IV and V genetic elements, and those carrying genes for PVL and *tst1* in the same cell. There was an increase in the proportion of isolates exhibiting resistance to multiple antibiotic classes. The emergence of multiple antibiotic resistant CC22-MRSA isolates poses challenges for effective empiric therapy and control of infections. These findings justify continuous monitoring of MRSA lineages to detect any changes in their virulence and antibiotic resistance profile for the benefit of effective control and prevention of their transmission.

## Data availability statement

The original contributions presented in the study are included in the article/Supplementary material; further inquiries can be directed to the corresponding author.

## Author contributions

EU: experimental design. SB and TV: data analysis. SB and EU: manuscript writing and editing. All authors contributed to the review of the article and approved the submitted version.

## Conflict of interest

The authors declare that the research was conducted in the absence of any commercial or financial relationships that could be construed as a potential conflict of interest.

## Publisher’s note

All claims expressed in this article are solely those of the authors and do not necessarily represent those of their affiliated organizations, or those of the publisher, the editors and the reviewers. Any product that may be evaluated in this article, or claim that may be made by its manufacturer, is not guaranteed or endorsed by the publisher.
