# Supplementary material for: Diversity of clonal complex 22 methicillin-resistant Staphylococcus aureus isolates in Kuwait hospitals
Source: Front Microbiol. 2022 Aug 4;13:970924. doi: 10.3389/fmicb.2022.970924 (PMC9386227; doi:10.3389/fmicb.2022.970924)
Supplement: Supplementary file 1 [file Data_Sheet_1.docx]

**Table S1.** Distribution of *spa* types among CC22-MRSA isolates from 2016 to 2018.

| Spa Type | 2016 | 2017 | 2018 | Total |
| --- | --- | --- | --- | --- |
| t223 | 43 | 55 | 62 | 160 |
| t032 | 18 | 15 | 27 | 60 |
| t852 | 22 | 20 | 17 | 59 |
| t005 | 29 | 19 | 8 | 56 |
| t309 | 10 | 5 | 15 | 30 |
| t14339 | 0 | 10 | 0 | 10 |
| t2834 | 0 | 4 | 2 | 6 |
| t790 | 5 | 3 | 1 | 9 |
| t8934 | 1 | 3 | 3 | 7 |
| t16605 | 1 | 4 | 1 | 6 |
| t3375 | 3 | 4 | 0 | 7 |
| t5844 | 0 | 5 | 0 | 5 |
| t8506 | 1 | 4 | 1 | 6 |
| t902 | 0 | 2 | 3 | 5 |
| t16202 | 2 | 0 | 4 | 6 |
| t2518 | 3 | 2 | 1 | 6 |
| t10028 | 2 | 2 | 0 | 4 |
| t1328 | 0 | 3 | 0 | 3 |
| t3107 | 1 | 2 | 1 | 4 |
| t5084 | 0 | 3 | 0 | 3 |
| t9606 | 0 | 2 | 1 | 3 |
| t016 | 1 | 1 | 0 | 2 |
| t10659 | 1 | 1 | 0 | 2 |
| t1317 | 3 | 1 | 0 | 4 |
| t14228 | 2 | 0 | 3 | 5 |
| t14230 | 1 | 1 | 1 | 3 |
| t1977 | 2 | 0 | 1 | 3 |
| t2336 | 0 | 0 | 2 | 2 |
| t2860 | 0 | 0 | 2 | 2 |
| t310 | 1 | 1 | 0 | 2 |
| t4018 | 0 | 2 | 0 | 2 |
| t541 | 1 | 0 | 1 | 2 |
| t5704 | 0 | 1 | 1 | 2 |
| t5708 | 1 | 2 | 0 | 3 |
| t6397 | 0 | 0 | 2 | 2 |
| t747 | 1 | 1 | 0 | 2 |
| t8221 | 2 | 0 | 1 | 3 |
| t845 | 2 | 0 | 1 | 3 |
| t022 | 0 | 0 | 1 | 1 |
| Spa Type | **2016** | **2017** | **2018** | **Total** |
| t10116 | 1 | 0 | 0 | 1 |
| t10118 | 1 | 0 | 0 | 1 |
| t10395 | 0 | 1 | 0 | 1 |
| t10405 | 1 | 0 | 0 | 1 |
| t1120 | 0 | 1 | 1 | 2 |
| t11618 | 0 | 0 | 1 | 1 |
| t11714 | 0 | 1 | 0 | 1 |
| t11836 | 1 | 1 | 0 | 2 |
| t12392 | 0 | 0 | 1 | 1 |
| t12413 | 0 | 1 | 0 | 1 |
| t12503 | 0 | 0 | 1 | 1 |
| t13024 | 0 | 0 | 1 | 1 |
| t13158 | 0 | 1 | 0 | 1 |
| t13697 | 0 | 1 | 0 | 1 |
| t14070 | 0 | 0 | 1 | 1 |
| t14392 | 0 | 1 | 0 | 1 |
| t15181 | 0 | 1 | 0 | 1 |
| t15203 | 0 | 0 | 1 | 1 |
| t1556 | 1 | 0 | 0 | 1 |
| t15801 | 1 | 0 | 0 | 1 |
| t1593 | 1 | 0 | 0 | 1 |
| t1612 | 0 | 0 | 1 | 1 |
| t16373 | 1 | 0 | 0 | 1 |
| t16468 | 1 | 0 | 0 | 1 |
| t16549 | 1 | 0 | 0 | 1 |
| t16578 | 1 | 0 | 0 | 1 |
| t16604 | 1 | 0 | 0 | 1 |
| t17200 | 0 | 0 | 1 | 1 |
| t2127 | 0 | 0 | 1 | 1 |
| t2251 | 1 | 0 | 0 | 1 |
| t2344 | 0 | 0 | 1 | 1 |
| t2424 | 0 | 0 | 1 | 1 |
| t2571 | 0 | 1 | 0 | 1 |
| t2597 | 1 | 0 | 0 | 1 |
| t2634 | 0 | 0 | 1 | 1 |
| t267 | 0 | 0 | 1 | 1 |
| t2672 | 0 | 1 | 0 | 1 |
| t2933 | 1 | 0 | 1 | 2 |
| t3010 | 1 | 0 | 0 | 1 |
| t311 | 0 | 0 | 1 | 1 |
| t3243 | 2 | 0 | 0 | 2 |
| Spa Type | **2016** | **2017** | **2018** | **Total** |
| t3304 | 0 | 0 | 1 | 1 |
| t3379 | 0 | 1 | 0 | 1 |
| t4326 | 3 | 0 | 0 | 3 |
| t4478 | 0 | 1 | 0 | 1 |
| t4565 | 1 | 1 | 0 | 2 |
| t4573 | 0 | 0 | 1 | 1 |
| t4892 | 1 | 1 | 0 | 2 |
| t5146 | 0 | 1 | 0 | 1 |
| t5307 | 0 | 0 | 1 | 1 |
| t5485 | 2 | 0 | 0 | 2 |
| t5634 | 1 | 1 | 0 | 2 |
| t5673 | 0 | 1 | 0 | 1 |
| t578 | 0 | 1 | 0 | 1 |
| t5995 | 0 | 0 | 1 | 1 |
| t6346 | 0 | 0 | 1 | 1 |
| t663 | 0 | 0 | 1 | 1 |
| t6827 | 1 | 0 | 0 | 1 |
| t688 | 0 | 0 | 1 | 1 |
| t7139 | 0 | 1 | 0 | 1 |
| t7604 | 1 | 0 | 0 | 1 |
| t8009 | 1 | 0 | 0 | 1 |
| t8962 | 0 | 1 | 0 | 1 |
| t9017 | 1 | 0 | 0 | 1 |
| t9411 | 0 | 0 | 1 | 1 |
| t9434 | 1 | 0 | 0 | 1 |
| t9448 | 0 | 1 | 0 | 1 |
| t9673 | 2 | 0 | 0 | 2 |
| t10347 | 0 | 1 | 0 | 1 |
| t2790 | 0 | 1 | 0 | 1 |
| ND | 3 | 25 | 27 | 55 |
| Total | **195** | **227** | **214** | **636** |

**Table S2.** Molecular characteristics of CC22-MRSA isolates

| **Genotypes (N)** | **Spa type (N)** | **Type IV subtype (N)** | **Sequence Type (ST)** | **PVL** | **TSST-1** | **Enterotoxins** | **Resistance genotype** |
| --- | --- | --- | --- | --- | --- | --- | --- |
| CC22-MRSA-[VI+fus] (n=4) | t16578 (1), t8934 (3) | VIg | 22, 2124 | - | + | egc | *vgaA, dfrS1, fusC* |
| CC22-MRSA-IV [tst1+/PVL+] (n=47) | t005 (16), t309 (10), t223 (6), t032 (2), t10659 (2), t10347 (1), t1120 (1), t2336 (1), t2790 (1), t5995 (1), ND (6) | IVa (23) | 22 | + | + | sec, sel, egc | *ermC, aacA-aphD, dfrS1, tetK* |
| CC22-MRSA-IV [fnbB-,sec/l-], UK-EMRSA-15/Barnim EMRSA (n=12) | t032 (5), t223 (2), t310 (2), t1612 (1), t16605 (1), t5084 (1) | IVa (5), IVh (1) | 22, 1037 | - | - | egc | *vgaA, dfrS1, ermC* |
| CC22-MRSA-IV [fnbB-,sec/l+], UK-EMRSA-15/Barnim EMRSA (n=22) | t032 (8), t747 (2), t790 (2), t223 (1), t13697 (1), t2634 (1), t267 (1), t7604 (1), t8506 (1), ND (4) | IVa (5), IVh (6) | 22 | - | - | seb, sec, sel, egc | *ermC* |
| CC22-MRSA-IV [fnbB+], UK-EMRSA-15/Barnim EMRSA (n=35) | t852 (15), t790 (7), t005 (2), t223 (2), t4892 (2), t309 (1), t13024 (1), t2672 (1), t4478 (1), t4326 (1), ND (2) | IVa (9), IVh (6) | 22, 2371 | - | - | sec, sel, egc | *ermC, aacA-aphD, aadD, dfrS1, tetK* |
| *****CC22-MRSA-IV [PVL+] (n=136) | t852 (42), t005 (31), t223 (7), t2518 (6), t902 (5), t3107 (4), ND (14) | IVa (15), IVh (15) | 22, 2286, 2371, 4671 | + | 6 | sea, sel, sek, seq, egc | *ermC, msr(A), mphC, aacA-aphD, aadD, aphA3, sat, dfrS1, fusB, tetK, cat, mupA, qacA, vga(A)* |
| **Genotypes (N)** | **Spa type (N)** | **Type IV subtype (N)** | **Sequence Type (ST)** | **PVL** | **TSST-1** | **Enterotoxins** | **Resistance genotype** |
| ******CC22-MRSA-IV [tst1+], UK-EMRSA-15/Middle Eastern variant (n=305) | t223 (134), t309 (17), t14339 (10), t3375 (7), t005 (6), t16202 (6), t16605 (5), t8506 (5), t2834 (5), t8934 (4), t5844 (4), t10028 (4), t9606 (3), t14228 (5), t5708 (3), t1977 (3), t8221 (3), t845 (3), ND (19) | IVa (219), IVh (1) | 22, 244, 1037, 1082 | - | + | sea, sec, sel, egc | *ermC, aacA-aphD, dfrS1, tetK, cat, vgaA* |
| CC22-MRSA-IV+V (n=61) | t032 (41), t223 (4), t022 (1), t17200 (1), t2344 (1), t2834 (1), t578 (1), t5844 (1), t9448 (1), ND (9) | IVa (7), IVh (24) | 22 | - | 9 | sea, seb, sec, sel, egc | *ermC, mupA, fusC, dfrS1, tet(M), fexA, sat* |
| CC22-MRSA-IV+V [PVL+] (n=7) | t032 (2), t309 (1), t852 (1), t005 (1), t15181 (1), ND (1) | IVa (1) | - | + | 4 | Sec, sel, egc | *ermC, aacA-aphD, aadD, dfrS1, tet(K), fusC, mupA, vgaA* |
| CC22-MRSA-V (n=2) | t2860 (2) |  | 737 | - | - | sea, egc | *tetK* |
| CC22-MRSA-V [fusC+] (n=3) | t233 (3) |  | 22 | - | + | egc | *fusc* |
| CC22-MRSA-IV [Q6GD50+], UK-EMRSA-15/Maltese variant (n=1) | t541 (1) | IVa (1) | - | - | - | egc | *fusc* |
| CC22-MRSA-[IV+fus+ccrAB4] (n=1) | t223 (1) | IV |  | + | + | sec, sel, egc | *ermC, aacA-aphD, dfrS1* |

***Spa* types found in two isolates:**

*****t016 (2), t4326 (2), t5485 (2). ****** t032 (2), t1328 (2), t14230 (2), t4018 (2), t4565 (2), t2933 (2), t3243 (2), t5084 (2), t5634 (2), t6397 (2), t9673 (2), t5704 (2).

***Spa* types found in single isolates:**

*****t309 (1), t11714 (1), t12392 (1), t1317 (1), t1328 (1), t14070 (1), t14230 (1), t14392 (1), t1593 (1), t3304 (1), t4573 (1), t5673 (1), t6827 (1), t688 (1), t8962 (1), t11836 (1), t16373 (1), t16468 (1), t16549 (1), t16604 (1), t8009 (1). ****** t15203 (1), t2127 (1), t2251 (1), t2336 (1), t2424 (1), t2571 (1), t3379 (1), t5146 (1), t5307 (1), t541 (1), t6346 (1), t663 (1), t7139 (1), t9017 (1), t9411 (1), t10116 (1), t10118 (1), t10405 (1), t1556 (1), t15801 (1), t2597 (1), t3010 (1), t9434 (1), t311 (1), t852 (1), t10395 (1), t1120 (1), t11618 (1), t11836 (1), t12413 (1), t12503 (1), t13158 (1).
